# Crystal structures of penicillin‐binding protein 3 in complexes with azlocillin and cefoperazone in both acylated and deacylated forms

**DOI:** 10.1002/1873-3468.12054

**Published:** 2016-01-23

**Authors:** Jingshan Ren, Joanne E. Nettleship, Alexandra Males, David I. Stuart, Raymond J. Owens

**Affiliations:** ^1^ Division of Structural BiologyHenry Wellcome Building for Genomic MedicineUniversity of OxfordOxfordUK; ^2^ OPPF‐UKThe Research Complex at HarwellRutherford Appleton LaboratoryOxfordshireUK; ^3^ Diamond Light SourcesHarwell Science and Innovation CampusDidcotUK

**Keywords:** azlocillin, cefoperazone, penicillin‐binding protein, *Pseudomonas aeruginosa*, thermal shift assay, β‐lactam antibiotics

## Abstract

Penicillin‐binding protein 3 (PBP3) from *Pseudomonas aeruginosa* is the molecular target of β‐lactam‐based antibiotics. Structures of PBP3 in complexes with azlocillin and cefoperazone, which are in clinical use for the treatment of pseudomonad infections, have been determined to 2.0 Å resolution. Together with data from other complexes, these structures identify a common set of residues involved in the binding of β‐lactams to PBP3. Comparison of wild‐type and an active site mutant (S294A) showed that increased thermal stability of PBP3 following azlocillin binding was entirely due to covalent binding to S294, whereas cefoperazone binding produces some increase in stability without the covalent link. Consistent with this, a third crystal structure was determined in which the hydrolysis product of cefoperazone was noncovalently bound in the active site of PBP3. This is the first structure of a complex between a penicillin‐binding protein and cephalosporic acid and may be important in the design of new noncovalent PBP3 inhibitors.

**Abbreviations**
ACA: anhydrodesacetyl cephalosporoic acid
AEC: acyl–enzyme complex
HMW: high molecular weight
LMW: low molecular weight
PBP3: penicillin‐binding protein 3
TG: transglycosylation
*T*_m_: melting temperature
TP: transpeptidase

Penicillin‐binding protein 3 (PBP3) of *Pseudomonas aeruginosa* is a membrane bound transpeptidase located on the periplasmic side of the inner membrane. It is involved in the synthesis of the peptidoglycan component of the bacterial cell wall by catalysing the cross‐linking of ᴅ‐alanine to form *N*‐acetylglucosamine‐*N*‐acetyl muramic polymers 1. PBP3 is a classified as a group B high molecular mass (HMM) enzyme lacking the glycosyltransferase activity of group A HMMs. The transpeptidase domains of both group A and B HMM enzymes are structurally similar to the lower molecular mass group C enzymes which are D carboxypeptidases responsible for the removal of terminal D‐alanine from the muramyl peptide. PBP3 and other penicillin‐binding proteins are inhibited by β‐lactam antibiotics which act as suicide substrates 2, 3 by mimicking the D‐alanyl‐D‐alanine stem peptide of the peptidoglycan precursors. β‐lactams block the activity of the transpeptidases and carboxypeptidases by acylating the active site serine residue 4. Resistance of pathogenic bacteria, including *P. aeruginosa*, to β‐lactam antibiotics has become a major issue 5, 6. There are a number of different resistance mechanisms including hydrolysis of the β‐lactam ring of antibiotics by β‐lactamases 7, increased expression of efflux pumps, reducing the permeability of the outer membrane and mutation of residues in the active site of PBPs 8, 9. The levels of expression of PBPs differ between β‐lactam resistant bacterial strains, however, this variation does not appear to be linked to resistance development 10.

Azlocillin (Fig. 1A) is representative of a group of ampicillin derivatives in which the ᴅ‐amino side group has been replaced with urea analogues and which are active against *P. aeruginosa* (MIC_50_ 50 μg·mL^−1^) 11. Cefoperazone (Fig. 1B), also known as cefobid, is a third‐generation cephalosporin 12 and is one of a small number of cephalosporins that are effective in treating Pseudomonas bacterial infections (MIC_50_ 25 μg·mL^−1^) 13. We have previously reported structures of PBP3 acyl–enzyme complexes (AECs) with β‐lactams of both penicillin and cephalosporin classes as well as a structure of PBP3 in complex with (*5S*)‐penicilloic acid (PA), a product of deacylated PBP3–piperacillin AEC 2, 14. In order to gain insight into the binding modes of azlocillin and cefoperazone, we have determined the crystal structures of PBP3 from *P. aeruginosa* in complexes with azlocillin and cefoperazone. The structure of the PBP3–azlocillin complex shows the ring‐opened β‐lactam intermediate covalently bound to S294. For the PBP3–cefoperazone complex, two different crystal structures were obtained. In one case, similar to the azlocillin complex, the cefoperazone (introduced by soaking) forms a covalent intermediate to the catalytic S294 of the active site. In the second structure, the ester‐linkage of cefoperazone with S294 is hydrolysed during the crystallization process and the product of the reaction, anhydrodesacetyl cephalosporoic acid (ACA), is observed bound in the active site. Similarities and differences between the three antibiotic bound structures and related ones are discussed.

**Figure 1:**
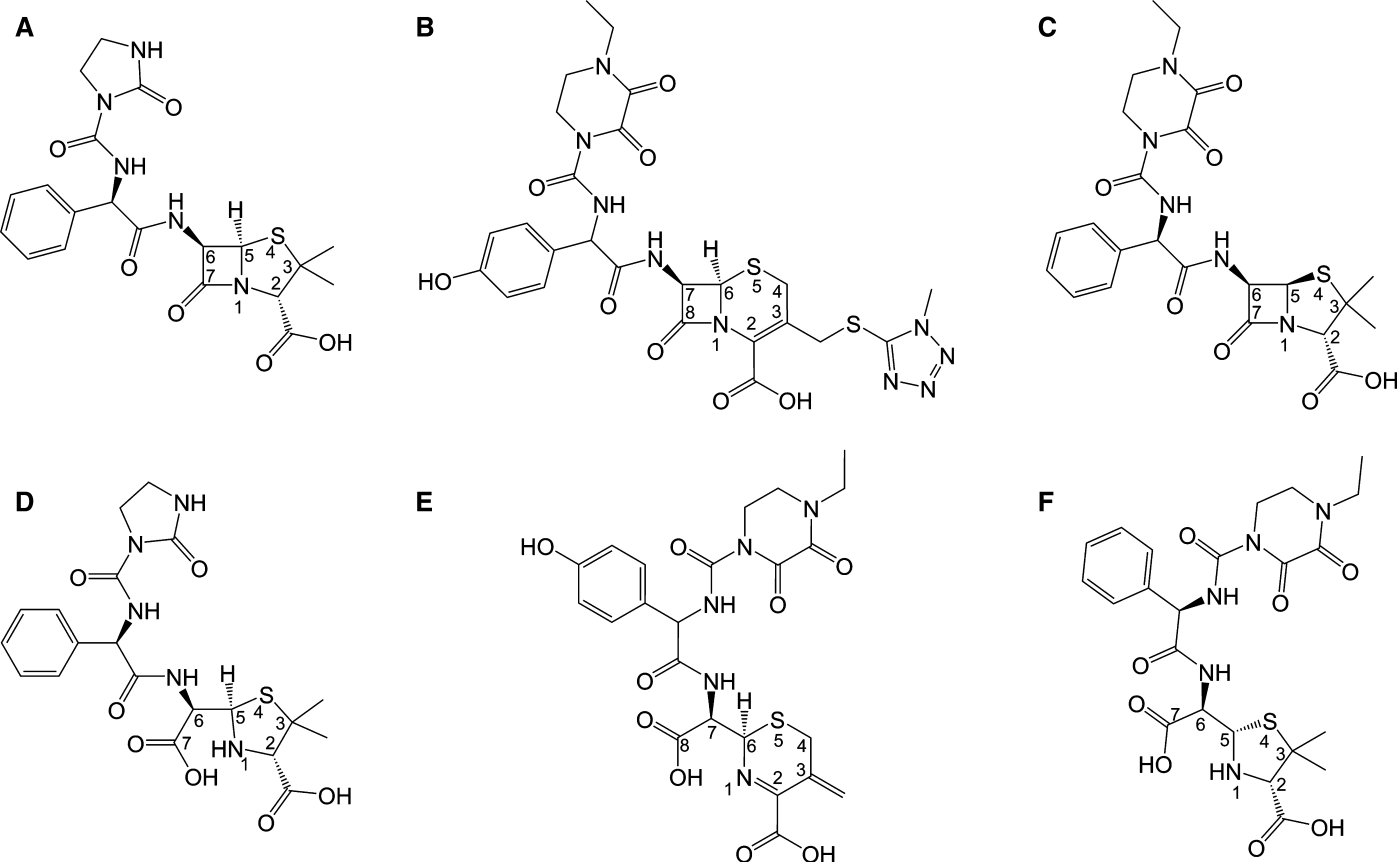
Chemical structures of β‐lactams and their hydrolysis products. (A) azlocillin, (B) cefoperazone and (C) piperacillin and their hydrolysis products (D), (E) and (F) respectively.

## Materials and methods

### Protein production and crystallization

The soluble domain of PBP3 (residues 35‐579) was produced using the method of Sainsbury *et al*. 2. Briefly, PBP3 inserted into the vector, pOPINF 15, which introduces a N‐terminal heaxahistidine tag separated by a rhinovirus 3C protease cleavage site, was expressed in *Escherichia coli* Rosetta^™^ 2(DE3) cells grown in autoinduction media 16. Cells were lysed and protein purified from the soluble fraction using nickel affinity followed by size exclusion chromatography in 20 mm Tris pH 7.5, 200 mm NaCl. Fractions containing PBP3 were combined and concentrated prior to use.

Crystallization screening experiments were performed with PBP3 at 4.3 mg·mL^−1^ in 200 nL volume sitting drops by vapour diffusion as previously described 17. Two types of PBP3 crystals were obtained, (a) in the presence of 0.5 mm azlocillin in a standard three row optimization of 2.5 m NaCl; 0.1 m imidazole pH 8.0 (Emerald Wizard 1 & 2 screen, condition D1) using the method of Walter *et al*. 17; (b) with the addition of 0.5 mm cefoperazone in 1.26 m (NH4)_2_SO_4_; 0.1 m CHES pH 9.5; 0.2 m NaCl (Emerald Wizard 1 & 2 screen, condition G5). Diffraction data were collected 9 days after the crystallization plates were set up. Data from a type (a) crystal showed no azlocillin at the active site of PBP3, while data from a type (b) crystal showed binding of the deacylated product of cefoperazone at the active site of PBP3. PBP3–azlocillin and PBP3–cefoperazone covalent complexes were then obtained by soaking the two penicillins separately with type (a) crystals for about 2 h at a concentration of 20 mg·mL^−1^, equating to 31 mm cefoperazone and 43 mm azlocillin respectively.

### Data collection and structure determination

X‐ray diffraction data were collected at 100K at beam lines I04‐1 and I02, Diamond Light Source. Diffraction images were indexed, integrated and merged with HKL2000 18. The structures were solved using molecular replacement program MOLREP 19 implemented in CCP4 suite 20 and coordinates of the PBP3–piperacillin complex 14 (PDB code 4KQO) as search model. Structures were refined with PHENIX 21 and model rebuilding was performed with COOT 22. The coordinates and structure factors have been deposited in the Protein Data Bank under accession numbers 5DF7 (PBP3–azlocillin AEC), 5DF8 (PBP3–cefoperazone AEC) and 5DF9 (PBP3–ACA complex).

### Thermal shift assay

Five microlitre of 20× SYPRO orange dye was added to 15 μL of protein at 4 mg·mL^−1^ along with 3 μL of 10 mm antibiotic solution. The sample was made up to 50 μL with 20 mm Tris pH 7.5 and 200 mm NaCl, sealed and heated in an Mx3005p qPCR machine (Stratagene, Agilent Technologies, USA) from 25 to 95 °C at a rate of 1 °C·min^−1^ 23. Fluorescence changes were monitored with excitation and emission wavelengths of 492 and 610 nm respectively. Reference wells, i.e. solutions consisting only of PBP3 and the dye, were used to compare the melting temperature (*T*_m_) values. Experiments were carried out in triplicate and *T*_m_ values were calculated for each well and compared to the reference *T*_m_ values to obtain Δ*T*_m_ for each compound.

## Results and Discussion

### The structure of the PBP3–azlocillin complex

**Figure 2:**
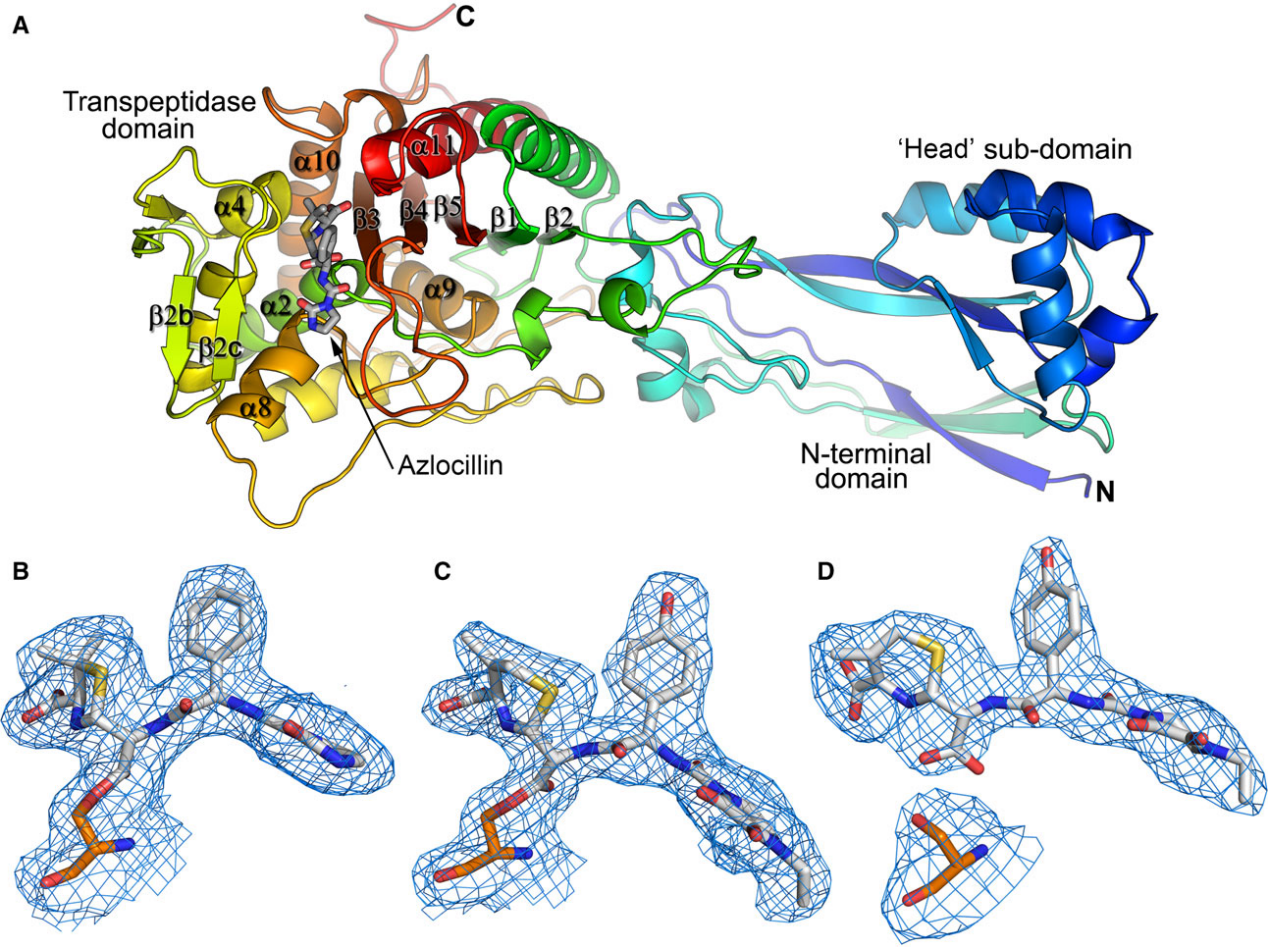
Overall structure of PBP3 and electron density maps. (A) Rainbow coloured cartoon representation of azlocillin PBP3 AEC, showing the overall structure of PBP3 and penicillin‐binding site. (B–D) *F*_*o*_ *‐F*_*c*_ omit electron density maps contoured at 3σ showing covalent binding of (B) azlocillin and (C) cefoperazone to give acyl–enzyme complexes, and noncovalent binding of (D) anhydrodesacetyl cephalosporoic, the product of deacylated cefoperazone, at the active site of PBP3. The antibiotics are shown with grey bonds and S294 is shown with orange bonds.

PBP3 (residues 35‐579) was produced as previously described 2. Attempts to produce crystals of the PBP3–azlocillin complex by cocrystallization were unsuccessful. Diffraction data collected from crystals, 9 days following setting up drops of protein and azlocillin showed no binding of the β‐lactam in the active site (data not shown). The structure was essentially identical to the apo structure reported previously 2, 8. Diffraction data were then collected from crystals that had additionally been soaked with azlocillin prior to data collection, and the electron density map showed clearly that the azlocillin had formed an acyl–enzyme complex (AEC) with PBP3 (Fig. 2B). The structure of PBP3 (Fig. 2A) can be divided into two domains, an N‐terminal domain (residues 35‐218) and a transpeptidase domain (residues 219‐579) (Fig. 2A). The N‐terminal domain is very flexible, often associated with weak electron density, especially for the loop regions in all our PBP3 structures of three different crystal forms 2, 14. A helical region towards the N terminus, called the head subdomain, is highly conserved among class B PBPs and is thought to be involved in cell wall synthesis through interactions with parts of the divisome 24. The N‐terminal domain also plays a role in stabilizing the folding of the transpeptidase domain 25. The transpeptidase domain folds to produce a central core of five anti‐parallel β‐sheets, β1‐β5, with β3 lining the top of the active site and β4, β2c, α4, α5, α8 and α11 (Fig. 2A) surrounding the substrate‐binding cleft which is wide enough to accommodate two peptidoglycan stems.

**Figure 3:**
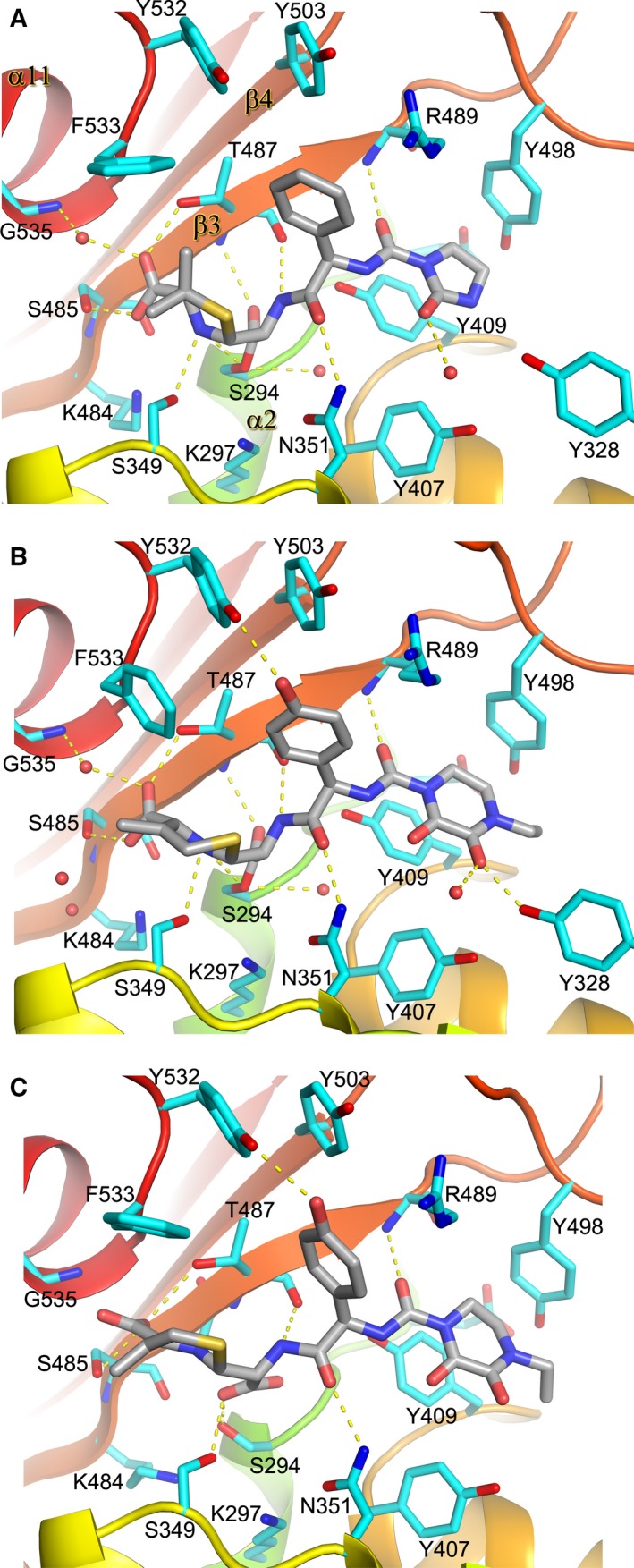
Details of protein‐inhibitor interactions. (A) azlocillin and (B) cefoperazone PBP3 covalent complexes, (C) PBP3–anhydrodesacetyl cephalosporoic (ACA) noncovalent complex. The protein backbone is shown as ribbons in rainbow colours from the N to C terminus. Key protein side‐chains are drawn as cyan sticks. The antibiotics are shown as grey sticks with nitrogen atoms in blue, oxygen atoms in red and sulfur atoms in yellow. The red spheres are water molecules. Potential hydrogen bonds are indicated by yellow dashed lines.

**Figure 4:**
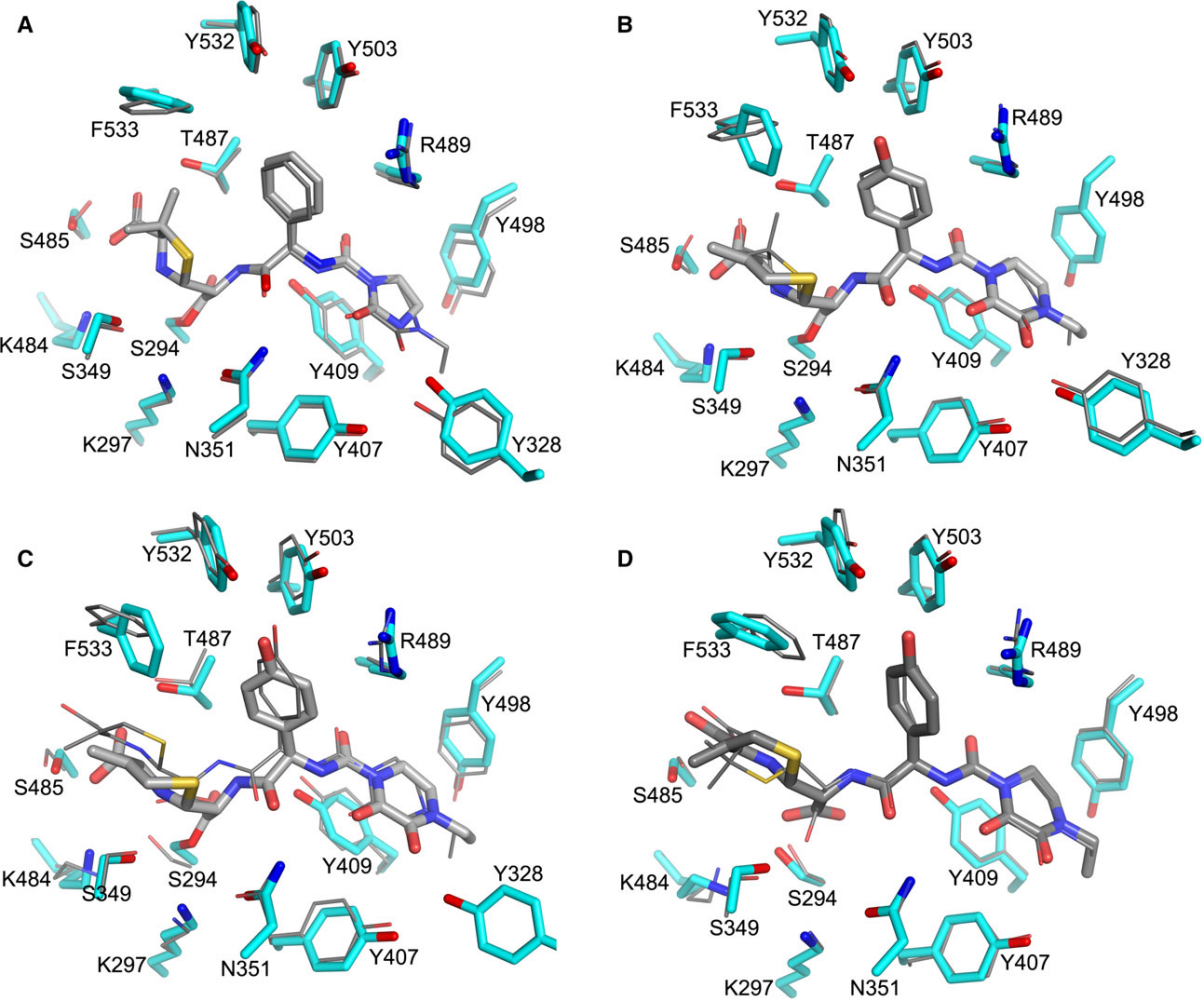
Comparison of covalent and noncovalent PBP3 complexes (A) Superposition of PBP3–azlocillin and PBP3–piperacillin (PDB id 4KQO) covalent complexes, (B) PBP3–cefoperazone and PBP3–piperacillin covalent complexes. (C) PBP3–cefoperazone and PBP3–anhydrodesacetyl cephalosporoic (ACA) complexes and (D) PBP3–ACA and PBP3–PA (PDB id 4KQR) complexes. In (A–C), the protein side‐chains in the azlocillin and cefoperazone acyl complexes are shown as thick cyan sticks and β‐lactams as thick grey sticks, the inhibitor and protein side‐chains in the piperacillin acyl complex and the PBP3‐ACA complex are shown as thinner sticks. The side‐chains and the inhibitor of PBP3‐ACA complex are shown as thick sticks in (D).

There are three conserved motifs found in the active site among all types of PBPs, SXXK, SXN and KSGT which are important in the catalysis 26. In PBP3, the SXXK motif is composed of S294, T295, V296 and K297, from the N terminus of α2 and located at the base of the cleft. In the PBP3–azlocillin AEC, the β‐lactam is covalently bound to the hydroxyl of the nucleophilic S294 via an ester‐linkage as observed in other PBP3–β‐lactam AECs 2. S349 and N351 from the SXN motif interact with the inhibitor via hydrogen bonds to the nitrogen of the thiazolidine ring and C‐9 carbonyl oxygen respectively (Fig. 3A). The third motif, KSGT, composed of residues 484‐487, orientates the thiazolidine ring by hydrogen binding the carboxylate group via the side‐chains of S485 and T487. The hydrogen bonds contributed from the backbone nitrogen and carbonyl oxygen of T487 and the nitrogen of R489 to the carbonyl oxygen of the ester‐linkage, N8 amino group and C12 carbonyl oxygen of the inhibitor, respectively, mimic the interactions between two antiparallel β‐strands as observed in the piperacillin acyl–enzyme complex 14 (Fig. 4A). In fact, the binding mode of azlocillin is very similar to that of piperacillin which also has a thiazolidine ring. However, the smaller oxoimidazolidin ring in azlocillin lacks the direct hydrogen bond to Y328 and makes weaker ring‐stacking interactions with Y409 and Y498 compared to the dioxopiperazine of piperacillin (PDB 4KQO and Fig. 4A).

### Structure of the PBP3–cefoperazone complex

The structure of PBP3–cefoperazone covalent complex was obtained by soaking a type (a) crystal with cefoperazone prior to data collection (Table 1). Examination of the acyl complex structure shows that the 1‐methyl‐5‐thiotetrazole group 27 of cefoperazone (Fig. 1) has been released during the catalytic opening of the β‐lactam ring (Fig. 2C). This leaves a double bonded methylene group on the end of the thiazine ring which is observed in the structure of PBP3 with well‐defined electron density (Fig. 2C). The release of 1‐methyl‐5‐thiotetrazole into the bloodstream following administration of cefoperazone can lead to hypoprothrombinemia. This is the result of the inhibition by 1‐methyl‐5‐thiotetrazole of glutamic acid γ‐carboxylation, a vitamin K‐dependent reaction required for the formation of active clotting factors 28.

**Table 1:** X‐ray data collection and refinement statistics

Data collection			
Data set	PBP3–azlocillin	PBP3–cefoperazone	PBP3–ACA
X‐ray source	Diamond I04‐1	Diamond I04‐1	Diamond I02
Wavelength (Å)	0.91730	0.91730	0.97960
Space group	*P1*	*P1*	*C2*
Cell dimensions (*a*,* b*,* c* (Å), α*,* β*,* γ (°))	57.3, 74.9, 82.7, 71.3°, 86.0°, 85.7°	57.2, 74.4, 82.4, 71.7°, 86.1°, 85.9°	176.9, 41.3, 87.8, 90°, 117.4°, 90°
Resolution (Å)	50.0–2.00 (2.07–2.00)	50.0–2.00 (2.07–2.00)	30.0–2.70 (2.80–2.70)
Unique reflections	76 005 (6889)	80 608 (7921)	15 866 (1552)
*R*_merge_	0.137 (0.579)	0.190 (0.641)	0.092 (0.722)
*I*/σ*I*	7.4 (1.6)	6.0 (1.7)	14.5 (1.9)
Completeness (%)	86.7 (78.3)	92.8 (90.7)	100 (100)
Redundancy	2.7 (2.4)	3.4 (2.9)	3.7 (3.7)
Refinement			
Resolution (Å)	50.0–2.00	50.0–2.00	50.0–2.70
No. reflections	72 141/3800	76 519/4066	15 052/789
R factor: (*R*_work_/*R*_free_)^a^	0.198/0.237	0.226/0.263	0.202/0.249
No. of atoms (protein/water/other)	7644/411/96	7638/605/110	3908/25/38
B‐factors (Å^2^) (protein/water/other)	47/44/42	35/39/33	66/49/75
R.m.s. deviations			
Bond lengths (Å)	0.006	0.007	0.004
Bond angles (°)	1.1	1.1	0.9
Ramachandran Plot			
Favoured (%)	90.7	90.6	86.8
Allowed (%)	9.3	9.4	12.7
Outliers (%)	0	0	0.5

a *R*_work_ and *R*_free_ are defined by *R* = Σ_*hkl*_||*F*_obs_| − |*F*_calc_||/Σ_*hkl*_|*F*_obs_|, where *h,k,l* are the indices of the reflections (used in refinement for *R*_work_; 5%, not used in refinement, for *R*_free_), *F*_obs_ and *F*_calc_ are the structure factors, deduced from measured intensities and calculated from the model respectively.

The active site structure of PBP3–cefoperazone covalent complex is very similar to that of PBP3–azlocillin, with the positions of Cα atoms of the key active site residues varying by less than 0.2 Å between the two acyl complexes (Fig. 3A,B), and most of the protein‐inhibitor hydrogen bond interactions are conserved between them (Table 2). However, there are key differences that reflect the different chemical structures of the two antibiotics (Fig. 1). The six‐membered thiazine ring derived from cefoperazone has a methylene group at the C‐3 position rather than the dimethyl group of the penicillin‐derived five‐membered thiazolidine ring in azlocillin. This methylene group interacts with the side‐chain of F533 so that the phenyl ring of F533 rotates by ~ 70° towards the methylene group. This is not observed in the azlocillin acyl–enzyme complex (Fig 3A). Cefoperazone also has a hydroxyphenyl ring enabling hydrogen bonding to Y532, whereas azlocillin has a phenyl ring and so it does not interact with the hydroxyl of Y532. Cefoperazone has a larger dioxopiperazine ring, in contrast to the oxoimidazolidin ring in azlocillin, making tighter off‐centre parallel and T‐shaped ring‐stacking interactions with Y409 and Y498 respectively (Fig. 3B). In addition, the larger size of the dioxopiperazine ring means that one of its oxygen atoms can form a direct hydrogen bond to the hydroxyl group of Y328 (2.9 Å). The ring structure of the β‐lactam at this position in both structures stabilizes Y498 and the whole β3‐β4 loop which are disordered in all published PBP3 covalent complexes with smaller β‐lactams 2, 8. Cefoperazone is closely related in structure to piperacillin principally differing as with azlocillin in the lactam ring (Fig 1). Superimposition of the covalent complexes between PBP3–cefoperazone and PBP3–piperacillin (PDB id 4KQO) shows that the two acyl complexes are very similar (Fig. 4B). The main difference is the orientation of F533 which is rotated in the cefoperazone complex compared to both the piperacillin and azlocillin covalent complexes (Fig. 4A). As previously described for other β‐lactams, covalent binding of azlocillin and cefoperazone is associated with a narrowing of the substrate‐binding cleft. Comparing both the azlocillin and cefoperazone PBP 3 acyl complexes with previously published structures 2, 8, 14 shows that a common set of residues are involved in the biding of a different β‐lactams to PBP3 (Table 2).

**Table 2:** Summary of hydrogen bond interactions in PBP3–β‐lactam AECs and deacylated product complexes

Inhibitor	Hydrogen bonds (≤ 3.2 Å)	PDB id	Reference
Carbenicillin	N‐S294, OG‐S349, ND2‐N351, OH‐Y409, OG‐S485, OG1‐T487, N‐T487, O‐T487	3OCL	Sainsbury *et al*. 2
Azlocillin	N‐S294, OG‐S349, ND2‐N351, OH‐Y409, OG‐S485, OG1‐T487, N‐T487, O‐T487, N‐R489	5DF7	This paper
Piperacillin	N‐S294, OH‐Y328, OG‐S349, ND2‐N351, OH‐Y407, OH‐Y409, OG‐S485, OG1‐T487, N‐T487, O‐T487, N‐R489	4KQO	van Berkel *et al*. 14
Cefoperazone	N‐S294, OH‐Y328, OG‐S349, ND2‐N351, OH‐Y409, OG‐S485, OG1‐T487, N‐T487, O‐T487, N‐R489, OH‐Y532	5DF8	This paper
Ceftazidime	OE2‐E291, N‐S294, OG‐S349, ND2‐N351, NZ‐K484, OG‐S485, OG1‐T487, N‐T487, O‐T487, N‐R489, O‐R489, NH1‐R489	3OCN	Sainsbury *et al*. 2
Aztreonam	OE1‐E291, N‐S294, OG‐S349, ND2‐N351, S485, NZ‐K484, OG1‐T487, N‐T487, O‐T487, NE‐R489	3PBS	Han *et al*. 8
ACA	OG‐S294, OG‐S349, ND2‐N351, OH‐Y409, OG‐S485, OG1‐T487, O‐T487, N‐R489, OH‐Y532, N‐G535	5DF9	This paper
(*5S*)‐PA	OG‐S294, OG‐S349, ND2‐N351, OH‐Y407, OH‐Y409, OG‐S485, OG1‐T487, N‐T487, O‐T487, N‐R489, N‐G535	4KQR	van Berkel *et al*. 14

### Structure of the PBP3–anhydrodesacetyl cephalosporoic acid complex

Cocrystallization of PBP3 with cefoperazone produced crystals of the deacylated product of the antibiotic, anhydrodesacetyl cephalosporoic acid (ACA), bound in the active site (Figs 2D and 3C). Comparison of the structures of the PBP3–cefoperazone covalent complex and the product complex showed that deacylation of the S294 does not affect the overall structure of PBP3, in particular the transpeptidase domain. In fact, the conformation of only a few key and conserved residues in the active site is altered between the two complexes (Fig. 4C). Notably, the position of the thiazine ring has changed significantly upon deacylation, shifted by ~ 2 Å away from S294 (Fig. 4C), whereas the piperazine ring remains almost unchanged. The movement of the thiazine ring is associated with rotation of F533 by about 50° anticlockwise. This avoids clashes and enables the carboxylate (which has rotated 90°) to cap the N terminus of α11 via a direct hydrogen bond to the amino group of G535 rather than via a water molecule as seen in the covalent complex (Fig. 3B). The side‐chain of S294 rotated 120° upon deacylation, makes bifurcated hydrogen bonds to the side‐chain of K484 and the carbonyl oxygen of S485 while maintaining a tight hydrogen bond (2.7 Å) to the carboxylate formed by ester hydrolysis of the covalent complex. This newly formed carboxylate is rotated clockwise by 45° into the same plane as the thiazine ring and no longer makes a hydrogen bond to the amino group of T487. The ethyl group on the dioxopiperazine ring has rotated 55° towards Y328, the side‐chain of which becomes disordered (Fig. 3B,C).

### Comparison of PBP3–anhydrodesacetyl cephalosporoic and PBP3–penicilloic complexes

There is one other report of PBP3 from *P. aeruginosa* noncovalently bound to a deacylated β‐lactam product, namely (*5S*)‐penicilloic acid (PA). In this case the complex was obtained by both cocrystallization of PBP3 with piperacillin and crystal soaking with the reaction product of piperacillin and a metallo‐β‐lactamase. In the PBP3–ACA structure, the thiazine ring is a (6*R*) epimer (Figs 1 and 4D) consistent with all other reported PBP3–β‐lactam covalent complexes 2, 8, 14. By contrast, the thiazolidine in the PBP3–PA noncovalent complex is the (5*S*) epimer (Figs 1 and 4D). It was previously shown that hydrolysis of piperacillin catalysed either by PBP3 or a metallo‐β‐lactamase initially results in (5*R*)‐PA, which was gradually converted to (5*S*)‐PA 14. Therefore, in the crystallographic experiments both enantiomers of PA would have been present. However, only (5S)‐PA was seen in the crystal structure. In the case of cefoperazone, only the R‐ enantiomer of ACA was observed noncovalently bound to PBP3. This indicates that either the ACA has not epimerized during crystallization or that the R‐enantiomer preferentially binds to PBP3.

Comparing the noncovalent PBP3–ACA and PBP3–PA complexes indicates why the S‐enantiomer of PA is favoured over the R‐enantiomer. Thus, if the thiazolidine of PA were in a (5*R*) stereochemistry its dimethyl group would clash with F533, whereas (6R)‐ACA is accommodated. To avoid steric clashes, the epimerization of PA also results in rotation ~ 80° of the carboxylate produced from the cleavage of the ester‐linkage. In both the covalent PBP3–cefoperazone and PBP3–ACA noncovalent complexes, the inhibitor makes addition hydrogen bonds compared to piperacillin and PA due to differences in substituents. The hydroxyphenyl side‐chain of ACA/cefoperazone forms a hydrogen bond with Y532 (Fig. 3B,C). In piperacillin and PA, the hydroxyphenyl is replaced with a phenyl side group and thus lacks this hydrogen bond interaction (Fig. 4B,D).

The observations that cocrystallization of PBP3 with azlocillin did not give a complex and the acyl complex could be obtained by crystal soaking suggest that the azlocillin covalent complex can be deacylated by PBP3. Interestingly, it did not prove possible to capture the structure of PBP3 with the ester‐linkage cleaved product of azlocillin presumably due to more rapid dissociation of the product compared to piperacillin and cefoperazone. This could be due to the dimethyl group on the thiazolidine ring and its smaller oxoimidazolidin group which reduce the affinity of the product to PBP3.

Examination of both noncovalent complexes suggests that binding could be increased by building into pockets in the structure. Thus, next to the methylene group of the ACA thiazine ring (dimethyl group of PA thiazolidine ring), a large space surrounded by N terminus of α11 and C‐termini of α4 and α10 is not occupied. This space can be targeted by addition of a chemical group to the thiadine ring of ACA or the thiazolidine ring of PA to increase the inhibitor interactions with PBP3 to make more potent non‐β‐lactam inhibitor.

### Thermal shift assay

**Figure 5:**
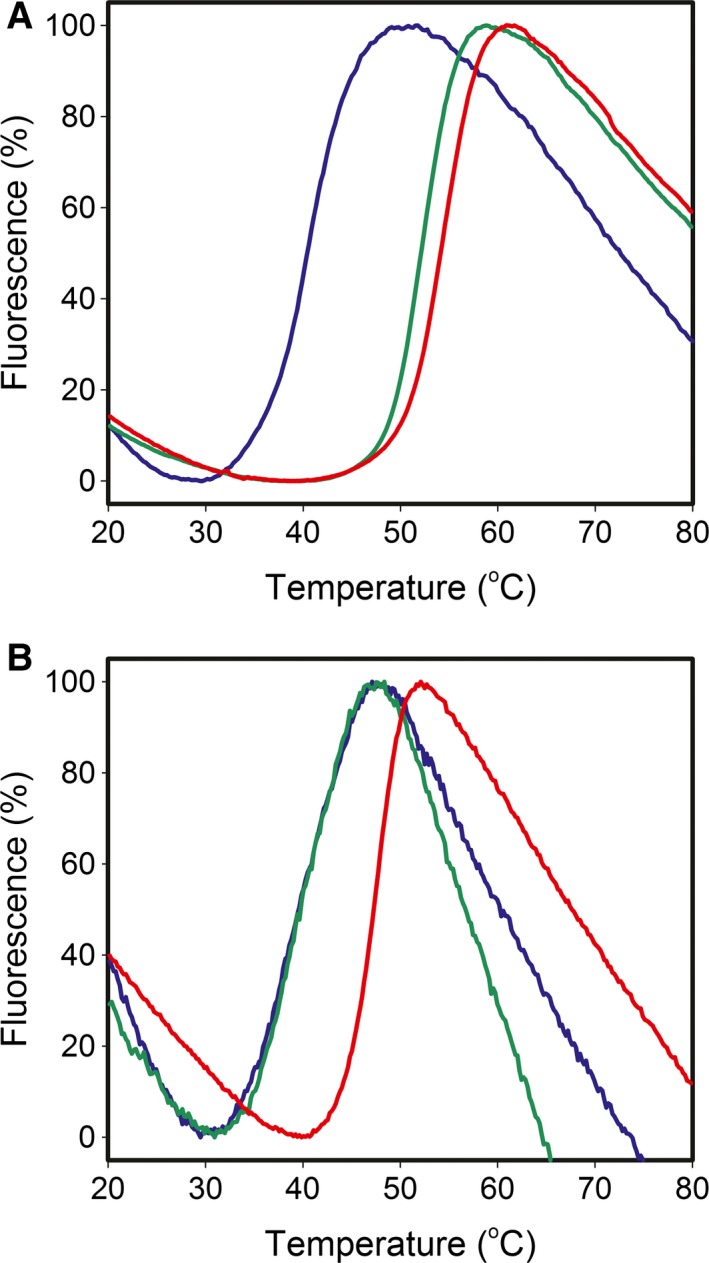
Thermal shift assay. Melt curves to compare the melting temperatures of both apo (A) PBP3 wild‐type and (B) PBP3 S294A with azlocillin and cefoperazone. (A) apo PBP3 blue, *T*_m_ of 40 ± 1 °C; PBP3 and azlocillin, green, *T*_m_ of 52 ± 1 °C; PBP3 and cefoperazone, red, *T*_m_ of 55 ± 1 °C; PBP3 (B) apo PBP3 S294A, blue, *T*_m_ of 39 ± 1 °C; PBP3 S294A and azlocillin, green, *T*_m_ of 39 ± 1 °C; PBP3 S294A and cefoperazone, red, *T*_m_ of 48 ± 1 °C.

A thermal shift assay was used to assess the effect of cefoperazone and azlocillin on the melting temperature (*T*_m_) of PBP3. The *T*_m_ of PBP3 alone was 40 ± 1 °C (Fig. 5A). Addition of either 0.6 mm azlocillin or 0.6 mm cefoperazone resulted in positive shifts in the *T*_m_ of 12 ± 1 °C and 15 ± 1 °C respectively (Fig. 5). To investigate whether this significant increase in *T*_m_ resulted from the formation of the acyl complex between PBP3 and the β‐lactams, the active site serine (S294) was changed to alanine and the mutated protein evaluated in the thermal shift assay. The results showed that preventing formation of the acyl complex by the S294A mutation, abolished the thermal shift due to addition of azlocillin (Fig. 5B). Thus, stabilization of PBP3 by this penicillin is entirely dependent on the formation of the acyl intermediate with S294. By contrast, a *T*_m_ shift of 9 ± 1 °C was observed for S294A PBP3 in the presence of cefoperazone (Fig. 5B) indicating that other interactions in addition to the formation of an acyl complex contribute to the stabilizing effect of the compound on PBP3 (Table 2). These observations are consistent with crystallographic results obtained with PBP3 and cefoperazone for which both covalent and noncovalently bound complexes were observed.

## Conclusion

Structural analysis of PBP3 from *P. aeruginosa* in complex with different β‐lactams (penicillins, carbapenems and cephalosporins) shows that a common set of residues, including the active site serine which becomes acylated, are key for binding. Additional hydrogen bond interactions contribute to the binding of different β‐lactams as determined by their substituents on the β‐lactam core. In all cases binding of the inhibitors is associated with a narrowing of the substrate‐binding cleft compared to the unliganded enzyme. Deacylation of the active site serine occurs over time and the crystal structures of the PBP3–ACA and PBP3–PA show that some inhibitors remain bound in the substrate‐binding cleft of the enzyme. This raises the possibility of using this structural information in the design of new nonlactam inhibitors which would not be susceptible to degradation by beta‐lactamases and hence overcome at least one mechanism of antibiotic resistance in the treatment of *Pseudomonas* infections.

## Author contributions

JR, JN, DIS and RJO devised the study, JN and AM purified and crystallized the PBP3 complexes, JR solved the structures of the PBP3 complexes. JR and RJO analysed the results and all authors contributed to writing the paper.
